# Psychotic depression and the risk of death due to suicide

**DOI:** 10.1192/j.eurpsy.2023.951

**Published:** 2023-07-19

**Authors:** T. Paljarvi, J. Tiihonen, M. Lähteenvuo, A. Tanskanen, S. Fazel, H. Taipale

**Affiliations:** 1 Department of Forensic Psychiatry, University of Eastern Finland, Kuopio, Finland; 2 Department of Clinical Neuroscience, Karolinska Institutet, Stockholm, Sweden; 3 Department of Psychiatry, Oxford University; 4 Warneford Hospital, 4Oxford Health NHS Foundation Trust, Oxford, United Kingdom; 5 School of Pharmacy, University of Eastern Finland, Kuopio, Finland

## Abstract

**Introduction:**

Depression markedly increases the risk of suicide, and depression is the most common psychiatric disorder diagnosed in persons with a completed suicide, but the interplay between depression and psychotic symptoms in suicides has remained unsettled.

**Objectives:**

The purpose of this study was to establish the risk of suicide associated with incident psychotic depression (PD) compared to incident non-psychotic severe depression (NPD) in a large nationwide cohort.

**Methods:**

This cohort study used routine data from nationwide health registers in Finland. Eligible participants were aged 18 – 59 years at the index diagnosis. Causes of death were defined by the International Classification of Diseases, 10th revision codes. The follow-up time was up to five years. Adjusted Cox regression models were used to analyse risk of death by method of suicide.

**Results:**

We included 17331 individuals with incident PD and 85989 individuals with incident NPD. Most of the deaths due to suicides occurred within the first two years after the index diagnosis. Compared to NPD, PD was associated with an overall two-fold increased risk of suicide (adjusted hazard ratio, (aHR) 2.19, 95% confidence interval (CI) 1.95, 2.46), after adjusting for psychiatric comorbidities. In PD, the highest relative risks were for impact-related suicides (aHR 3.03, 95%CI 2.23, 4.13) and for suffocation-related suicides (aHR 2.72, 95%CI 2.23, 3.30), whereas the lowest relative risk was for intentional poisonings (aHR 1.66, 95%CI 1.37, 2.02).

**Conclusions:**

Psychotic symptoms increased the risk of suicide 2-fold of the risk that was associated with severe depression, after controlling for comorbid psychiatric disorders. The severity of suicidal ideation may be higher in PD than in NPD, which then leads to more lethal methods of self-harm.

**Disclosure of Interest:**

T. Paljarvi: None Declared, J. Tiihonen Grant / Research support from: Janssen-Cilag, Eli Lilly, Consultant of: HLS Therapeutics, Orion, and WebMed Global, Speakers bureau of: Eli Lilly, Evidera, Janssen-Cilag, Lundbeck, Mediuutiset, Otsuka, Sidera, and Sunovion, M. Lähteenvuo Shareolder of: Genomi Solutions ltd, Nursie Health ltd, Springflux ltd, Grant / Research support from: Finnish Medical Foundation, Emil Aaltonen Foundation, Speakers bureau of: Sunovion, Lundbeck, Otsuka Pharma, Orion Pharma, Recordati, Janssen, Janssen-Cilag, A. Tanskanen Grant / Research support from: Janssen-Cilag, Eli Lilly, S. Fazel Grant / Research support from: Wellcome Trust, H. Taipale Grant / Research support from: Janssen-Cilag, Eli Lilly, Academy of Finland, Speakers bureau of: Janssen-Cilag, Otsuka

